# Overexpression of a *Prefoldin β* subunit gene reduces biomass recalcitrance in the bioenergy crop *Populus*

**DOI:** 10.1111/pbi.13254

**Published:** 2019-09-27

**Authors:** Jin Zhang, Meng Xie, Mi Li, Jinhua Ding, Yunqiao Pu, Anthony C. Bryan, William Rottmann, Kimberly A. Winkeler, Cassandra M. Collins, Vasanth Singan, Erika A. Lindquist, Sara S. Jawdy, Lee E. Gunter, Nancy L. Engle, Xiaohan Yang, Kerrie Barry, Timothy J. Tschaplinski, Jeremy Schmutz, Gerald A. Tuskan, Wellington Muchero, Jin‐Gui Chen

**Affiliations:** ^1^ Biosciences Division Oak Ridge National Laboratory Oak Ridge TN USA; ^2^ Center for Bioenergy Innovation Oak Ridge National Laboratory Oak Ridge TN USA; ^3^ Department of Plant Sciences University of Tennessee Knoxville TN USA; ^4^ Chemical & Biomolecular Engineering University of Tennessee Knoxville TN USA; ^5^ College of Textiles Donghua University Shanghai China; ^6^ ArborGen Inc. Ridgeville SC USA; ^7^ U.S. Department of Energy Joint Genome Institute Walnut Creek CA USA; ^8^ HudsonAlpha Institute for Biotechnology Huntsville AL USA

**Keywords:** *Populus*, prefoldin, biofuels, cell wall recalcitrance, lignin, S/G ratio, transcriptome, metabolome

## Abstract

Prefoldin (PFD) is a group II chaperonin that is ubiquitously present in the eukaryotic kingdom. Six subunits (PFD1‐6) form a jellyfish‐like heterohexameric PFD complex and function in protein folding and cytoskeleton organization. However, little is known about its function in plant cell wall‐related processes. Here, we report the functional characterization of a *PFD* gene from *Populus deltoides*, designated as *PdPFD2.2*. There are two copies of *PFD2* in *Populus,* and *PdPFD2.2* was ubiquitously expressed with high transcript abundance in the cambial region. PdPFD2.2 can physically interact with DELLA protein RGA1_8g, and its subcellular localization is affected by the interaction. In *P. deltoides* transgenic plants overexpressing *PdPFD2.2*, the lignin syringyl/guaiacyl ratio was increased, but cellulose content and crystallinity index were unchanged. In addition, the total released sugar (glucose and xylose) amounts were increased by 7.6% and 6.1%, respectively, in two transgenic lines. Transcriptomic and metabolomic analyses revealed that secondary metabolic pathways, including lignin and flavonoid biosynthesis, were affected by overexpressing *PdPFD2.2*. A total of eight hub transcription factors (TFs) were identified based on TF binding sites of differentially expressed genes in *Populus* transgenic plants overexpressing *PdPFD2.2*. In addition, several known cell wall‐related TFs, such as *MYB3*,* MYB4*,* MYB7*,* TT8* and *XND1*, were affected by overexpression of *PdPFD2.2*. These results suggest that overexpression of *PdPFD2.2* can reduce biomass recalcitrance and *PdPFD2.2* is a promising target for genetic engineering to improve feedstock characteristics to enhance biofuel conversion and reduce the cost of lignocellulosic biofuel production.

## Introduction

A protein's function is partially controlled by its three‐dimensional structure and is dependent on a complex network of molecular chaperones (Kim *et al*., 2013). Molecular chaperones are crucial for cellular development and play important roles in protein assembly, folding, trafficking and degradation (Hartl *et al*., 2011). Prefoldin (PFD) was first described as a co‐chaperone protein and is ubiquitously present across the eukaryotic kingdom (Vainberg *et al*., 1998). PFD is now classified as a group II chaperonin, which is found in archaea and eukaryotes and appears to be central to a complex network of co‐chaperones. Eukaryotic PFD is polymeric and consists of six subunits: two α subunits (PFD3 and PFD5) and four β subunits (PFD1, PFD2, PFD4 and PFD6) (Siegert *et al*., 2000). These six subunits form a ‘jellyfish‐like’ heterohexameric complex, which can deliver newly synthesized unfolded protein to cytosolic chaperonins containing TCP‐1 for protein folding and protecting unfolded proteins in this process (Siegert *et al*., 2000).

Prefoldin plays a central role in cellular development and its function has been well studied from yeast to mammalian species. In yeast, PFD can bind to tubulin and actin to facilitate productive folding and binding inside the chaperonin cavity, and deletion of a single or multiple PFD subunits results in cytoskeleton disruption (Geissler *et al*., 1998). Reduction of PFDs causes *Caenorhabditis elegans* embryonic lethality, and silencing of PFD1, PFD2, PFD3 and PFD6 subunits reduces microtubule growth (Lundin *et al*., 2008). Human PFD inhibits amyloid‐β fibrillation and is involved in the nontoxic amyloid‐β aggregation, which is consistent with its role in Alzheimer's disease (Broer *et al*., 2011; Sörgjerd *et al*., 2013). Eukaryotic PFD is highly conserved, as evidenced by the fact that plant and human subunits can functionally complement yeast *pfd* mutants (Geissler *et al*., 1998; Rodriguez‐Milla and Salinas, 2009). In *Arabidopsis*, only one copy of *PFD* is present for each of the six subunits (Hill and Hemmingsen, 2001). Among these, PFD3, PFD5 and PFD6 are required for normal microtubule dynamics and organization (Gu *et al*., 2008; Rodriguez‐Milla and Salinas, 2009). The *Arabidopsis pfd6‐1* mutant exhibits a range of microtubule defects, including hypersensitivity to oryzalin, and defects in cell division, cortical array organization and microtubule dynamicity (Gu *et al*., 2008). In addition, *Arabidopsis pfd3* and *pfd5* mutants showed reduction of α‐ and β‐tubulin, alteration in developmental patterns and microtubule organization and hypersensitivity to salt stress (Rodriguez‐Milla and Salinas, 2009). As a fundamental component of plant cell wall, cellulose is synthesized by cellulose synthase (CESA) and is associated with the dynamics of the cytoskeleton via actin filaments and microtubules (Cai *et al*., 2011). During secondary cell wall formation, the cellulose synthase complex (CSC) has been observed to form bands beneath sites of secondary cell wall synthesis, which is dependent upon underlying bundles of microtubules and the thick actin cables (Paredez *et al*., 2006; Wightman and Turner, 2008). Therefore, PFD subunits may have a role in cell wall‐related processes, but this has been unstudied.

Besides the cytoskeletal complexes, PFD subunits can also assemble non‐cytoskeletal complexes in the cytoplasm. For instance, a PFD‐like complex participates in the cytoplasmic assembly of RNA polymerase II (Boulon *et al*., 2010) and in the stabilization and assembly of phosphatidylinositol 3‐kinase‐related kinases in cooperation with the R2TP complex (Horejsi *et al*., 2010). In addition, PFD is involved in hormonal signalling pathways. The PFD complex directly interacts with DELLA proteins, and its subcellular localization has been shown to be gibberellin (GA)‐dependent (Locascio *et al*., 2013). PFD also shuttles between the cytoplasm and the nucleus and acts on DNA‐binding proteins. In yeast, all of the PFD subunits can be detected in the nucleus (Millan‐Zambrano *et al*., 2013). An α‐class PFD‐like subunit, UXT, is also located in human centrosomes, associated with γ‐tubulin, and its overexpression disrupts the centrosome structure (Zhao *et al*., 2005). In plants, PFD5 and PFD6 are also found in the nucleus of tobacco and *Arabidopsis* leaf cells. Depending on the physical interaction with the nuclear DELLA proteins, the PFD complex can stay in the nucleus to affect microtubule orientation when GA is absent or can be localized in the cytoplasm when GA is present (Locascio *et al*., 2013). Localization of PFD in the nucleus may be the result of the regulation of its cytoplasmic functions by means of a cytoplasm‐exclusion mechanism, as has been demonstrated in *Arabidopsis* (Locascio *et al*., 2013). PFD has also been shown to play transcriptional roles in mammalian cells. For example, human PFD5 can interact with c‐Myc, EGR1 and p73 (Satou *et al*., 2001; Watanabe *et al*., 2002); PFD3 can interact with HIV integrase, hMSH4 and NF‐κB (Kim *et al*., 2008; Mousnier *et al*., 2007); another PFD‐like subunit UXT can bind to EVI1, NF‐κB, ALS2, LRP16, TAF130, Sp1, etc., to play regulatory roles (Millan‐Zambrano and Chavez, 2014). In plants, except for its interaction with DELLA in *Arabidopsis* (Locascio *et al*., 2013), little is known about the transcriptional or regulatory roles of PFD.

Presently, plant cell walls are considered to be a renewable resource for the conversion and production of biofuels (Pauly and Keegstra, 2010). The cell wall composition, that is, lignin, cellulose and hemicellulose content, and cell wall properties, for example, lignin monomer ratios and hemicellulose types, impact sugar release efficiency during biofuel conversion (Himmel *et al*., 2007; Zhang *et al*., 2019). However, the effect of monolignol composition on biomass digestibility varies greatly in different species or under different treatment conditions. For example, S/G ratio is negatively related to the enzymatic hydrolysis of untreated biomass of maize, poplar and eucalyptus. On the other hand, high S/G ratio can enhance saccharification of poplar and *Arabidopsis* biomass with hot water pretreatment (Li *et al*., 2010; Studer *et al*., 2011) and poplar biomass with steam explosion pretreatment (Mansfield *et al*., 2012). In this study, we describe the characterization of a *PFD β* subunit gene in *Populus deltoides*,* PdPFD2.2*, encoded by the locus Potri.008G153900. We overexpressed this gene in *P. deltoides* ‘WV94’ and found that it altered the lignin composition and sugar release characteristics in transgenic poplar plants. We also performed transcriptomic and metabolomic analyses to explore the molecular mechanism of changes in cell wall precursors. Our results suggest that PFD2.2 is likely involved in reactions leading to structural changes in cell wall composition. This study provides insights into the role of a prefoldin protein in the genetic control of the dynamic changes in cell wall composition, and provides a foundation for reducing cell wall recalcitrance in the bioenergy crop *Populus*.

## Results

### *PFD2* genes in *Populus*

**Figure 1:**
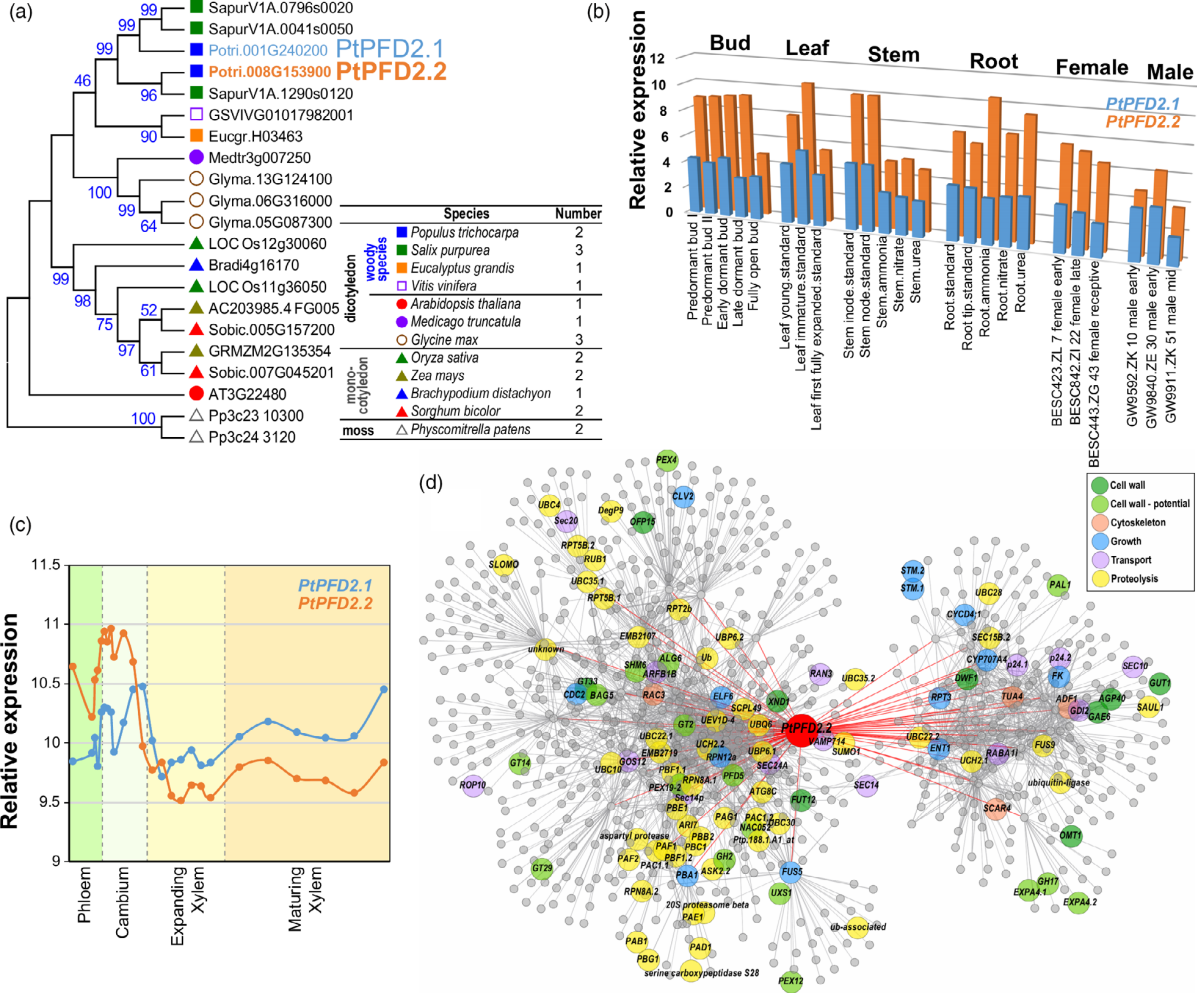
Phylogenetic analysis and expression patterns of the *Populus PFD2* genes. (a) Phylogenetic tree constructed using full‐length amino acid sequences of PFD2 from 12 plant species by the neighbour‐joining (NJ) method with 1000 bootstrap replicates. (b) Expression patterns of the *PtPFD2* genes across various tissues. (c) Expression patterns of the *PtPFD2* genes during wood formation. The data were retrieved from the AspWood database (http://aspwood.popgenie.org/aspwood-v3.0/), where relative expression is shown for aspen stem samples, which consist primarily of phloem, cambium, expanding xylem and maturing xylem. (d) Co‐expression network of *PtPFD2.2*.

To identify PFD2 proteins in *Populus*, we performed a Blastp search against the *P. trichocarpa* genome (V3.0) using *Arabidopsis* PFD2 (AT3G22840) as a query. Two copies of PFD2 (Potri.001G240200 and Potri.008G153900) were identified and named PtPFD2.1 and PtPFD2.2, respectively (Figure 1a), with both containing the Prefoldin_2 motif. To explore the evolutionary relationship of PFD2 in the plant kingdom, we compared PFD2 from 12 plant species, including four woody dicots: *Populus* (*P. trichocarpa*), *Salix* (*S. purpurea*), *Eucalyptus* (*E. grandis*) and grape (*Vitis vinifer*); three herbaceous dicots: *Arabidopsis* (*A. thaliana*), *Medicago* (*M. truncatula*) and soybean (*Glycin max*); four monocots: rice (*Oryza sativa*), maize (*Zea mays*), *Brachypodium distachyon* and *Sorghum* (*S. bicolor*); and one ancient nonvascular moss: *Physcomitrella patens* (Figure 1a). One to three PFD2 copies were identified from each of these 12 species. *Salix* and soybean contain three copies of PFD2, whereas *Arabidopsis*,* Medicago*,* Eucalyptus*, grape and *Brachypodium* contain only one copy in each genome. To examine the evolutionary relationships of PFD2 proteins across different plant species, we performed a maximum likelihood (ML) phylogenetic analysis using the full‐length amino acid sequences of PFD2 proteins. Except for *Arabidopsis* PDF2, the PFD2 genes from woody dicots, herbaceous dicots, monocots and moss were grouped into different clades (Figure 1a), which implies that their functions might have been diverged during evolution.

Next, we analysed expression patterns of the *Populus PFD2* genes across various tissues and organs in the *Populus* Gene Atlas (https://phytozome.jgi.doe.gov/phytomine/begin.do). Both *PtPFD2.1* and *PtPFD2.2* were highly expressed in stem (internode and node) and immature leaf (Figure 1b). Based on the AspWood gene expression database, which contains high‐resolution RNA‐Seq data from nanometre‐scale tissues of the *P. tremula* stem development, *PtPFD2.1* and *PtPFD2.2* were highly expressed in the cambium with decreased expression during xylem expansion, but increased again during xylem maturation (Figure 1c). Notably, *PtPFD2.2* has higher transcript abundance than *PtPFD2.1* in both the stem and cambium (Figure 1b, c). We then constructed a co‐expression network of *PtPFD2.2* based on global gene expression patterns across different tissues and under various stresses. Based on the functional classification, genes related to proteolysis (54 genes), cell wall (26 genes), transport (14 genes), growth (13 genes) and cytoskeleton (4 genes) were co‐expressed with *PtPFD2.2* (Figure 1d). GO enrichment analysis showed that ‘protein modification’‐related GO terms were significantly enriched in the network (Figure S1). To explore the potential regulatory mechanism of *PtPFD2.2* expression, we analysed the *cis*‐acting elements of the promoter region (3000 bp upstream of the translation initiation site) of *PtPFD2.2*. The identified *cis*‐acting elements were classified into four groups (development, hormone, stress and other) according to their potential functions. Notably, a total of six MYB binding sites, including three MBS (MYB binding site involved in drought inducibility), two MRE (MYB binding site involved in light responsiveness) and one CCAAT‐box (MYBHv1 binding site), were found in the promoter of *PtPFD2.2* (Figure S2). For hormone‐related *cis*‐elements, five gibberellin‐responsive elements (four GARE‐motifs and one P‐box) and four salicylic acid‐responsive elements (TCA‐elements) were identified (Figure S2).

### Protein structure of *Populus PFD2.2*

In order to understand the functional mechanism of PtPFD2.2, we analysed its protein structure. PtPFD2.2 is 145 aa in length. It has two predicted α‐helices connected by two β‐sheets, and the majority of the whole protein is constructed as the Prefoldin_2 motif (13‐118 aa; Figure S3). Based on the predicted posttranslation modification, a total of 20 phosphorylation, one sumoylation and four ubiquitination sites were identified. The phosphorylation sites were enriched in the first α‐helix and the C‐terminal tail. For ubiquitination sites, one is located in the first α‐helix and three in the C‐terminal tail (Figure S3). Three‐dimensional structure prediction by I‐TASSER analysis revealed that two alpha‐helices are in parallel arrangement.

### PFD2.2 physically interacts with DELLA protein in *Populus*

**Figure 2:**
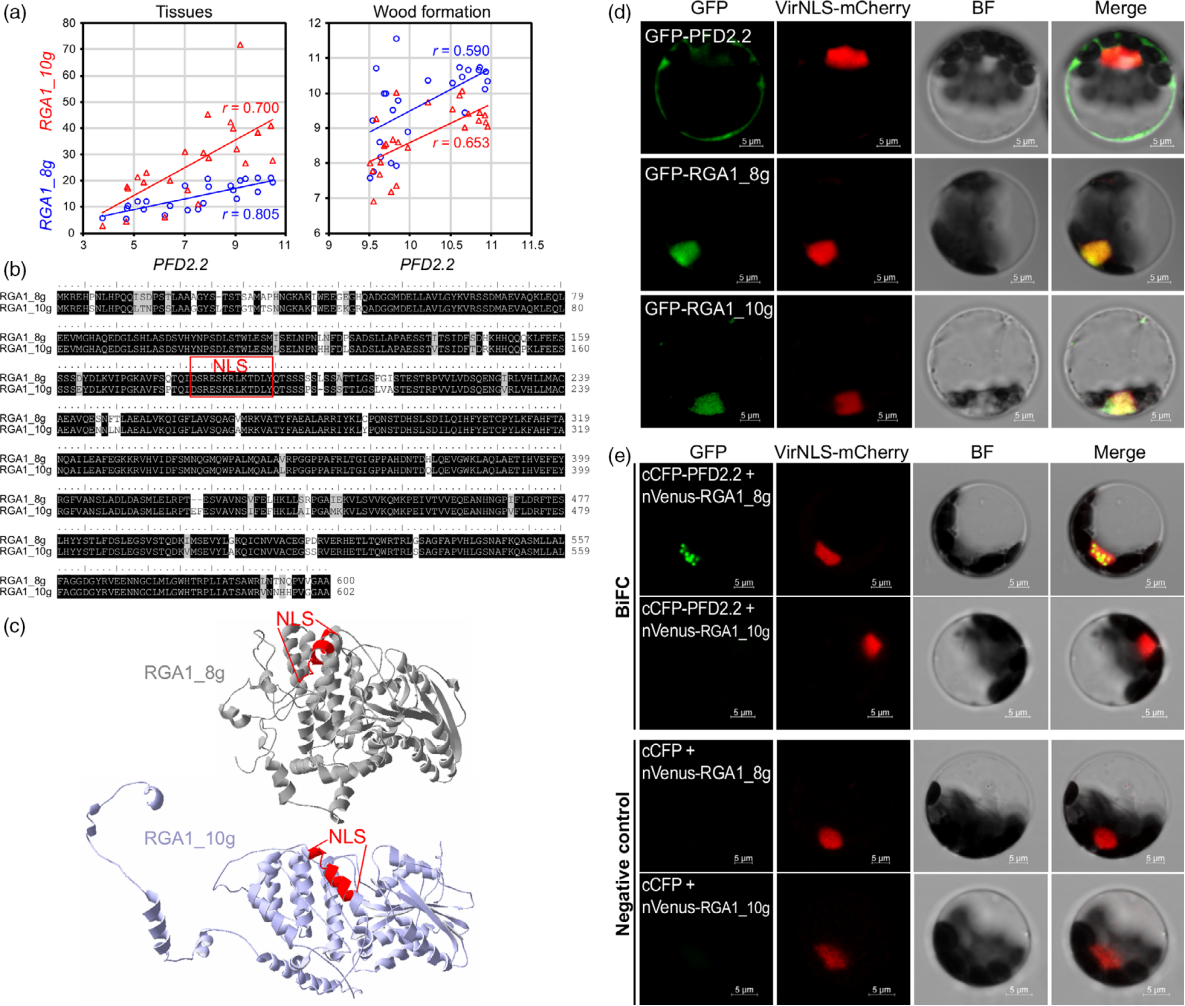
PFD2.2 physically interacts with a DELLA protein in *Populus*. (a) Expression correlation between *PFD2.2* and two *DELLA* genes (*RGA1_8g* and *RGA1_10g*) in tissue samples (24 samples including different developmental stages of bud, leaf, stem, root and female and male catkins) and wood formation samples (25 samples from phloem, cambium and expanding and maturing xylem). (b) Sequence alignment of RGA1_8g and RGA1_10g proteins and nuclear localization signal (NLS). (c) Protein structures of RGA1_8g and RGA1_10g. (d) Subcellular localization of PFD2.2, RGA1_8g and RGA1_10g in *Populus* protoplast (green). The nuclear marker mCherry‐VirD2NLS is shown in red. Bar = 5 μm. (e) Bimolecular fluorescence complementation (BiFC) assay of PFD2.2 and two DELLA proteins (RGA1_8g and RGA1_10g). The nuclear marker mCherry‐VirD2NLS is shown in red. Bar = 5 μm.

To explore whether the interaction between PFD and DELLA proteins is conserved in *Populus* as observed in *Arabidopsis*, we examined the protein‐protein interaction between PtPFD2.2 and PtDELLA proteins. First, we sought to identify the *Populus* DELLA homolog that may potentially interact with PtPFD2.2 by comparing the expression correlation with *PtPFD2.2* among four *DELLA* genes in the *Populus* genome. Within the *DELLA* gene family, two genes (*RGA1_8g* and *RGA1_10g*) showed high expression correlation with *PtPFD2.2* in both tissue samples and wood formation process (Figure 2a). RGA1_8g and RGA1_10g are paralogous, and both proteins contain a nuclear localization signal (NLS) peptide (Figure 2b). Although the two proteins showed high sequence similarity, their protein 3D structures were different due to the N‐terminal sequence divergence (Figure 2c).

To determine the subcellular localization of PFD2.2, RGA1_8g and RGA1_10g, each of these three proteins was fused with Yellow Fluorescent Protein (YFP) at their N‐terminus and were transiently expressed in the *Populus* leaf mesophyll protoplasts (Figure 2d). Signals of YFP‐RGA1_8g and YFP‐RGA1_10g (green colour) overlapped with the red signal of the nuclear marker mCherry‐VirD2NLS (Lee *et al*., 2008), indicative of the nuclear localization of RGA1_8g and RGA_10g. In contrast, PFD2.2 appeared to be localized outside of the nucleus, because the signal of YFP‐PFD2.2 had no overlap with the signal of mCherry‐VirD2NLS.

To investigate the *in vivo* association of PFD2.2 with RGA1_8g or RGA1_10g, we performed the bimolecular fluorescence complementation (BiFC) assay. In the BiFC assay, the PFD2.2 protein was fused with the C‐terminal fragment of cyan fluorescent protein (cCFP). RGA1_8g and RGA1_10g proteins were fused with the N‐terminal fragment of Venus (nVenus). We then introduced paired proteins into the *Populus* leaf mesophyll protoplasts by PEG‐calcium‐mediated transfection. The interaction of cCFP‐tagged protein with nVenus‐tagged protein is indicated by yellow fluorescence due to the formation of an intact YFP. As shown in Figure 2e, BiFC signals (green colour) resulted from PFD2.2 and RGA1_8g interaction were present as distinct nuclear speckles (shown as yellow colour). Such interaction was not detected in either the PFD2.2‐RGA1_10g pair or the negative controls. Collectively, these results indicate that RGA1_8g may associate with PFD2.2 and possibly guide PFD2.2 into the nucleus.

### Growth and cell wall components in *Populus* transgenic plants overexpressing *PdPFD2.2*

To characterize the function of *PFD2.2* in *Populus*, the full‐length CDS of *PFD2.2* was cloned from *P. deltoides* ‘WV94’ (*PdPFD2.2*) and overexpressed in ‘WV94’. From five independent transgenic lines, two lines that had relatively high *PdPFD2.2* expression levels were selected for further study and labelled as #1 and #2. The transcript level of *PdPFD2.2* was examined by qRT‐PCR and both *PdPFD2.2* transgenic lines were confirmed to overexpress *PdPFD2.2* (Figure S4).

**Figure 3:**
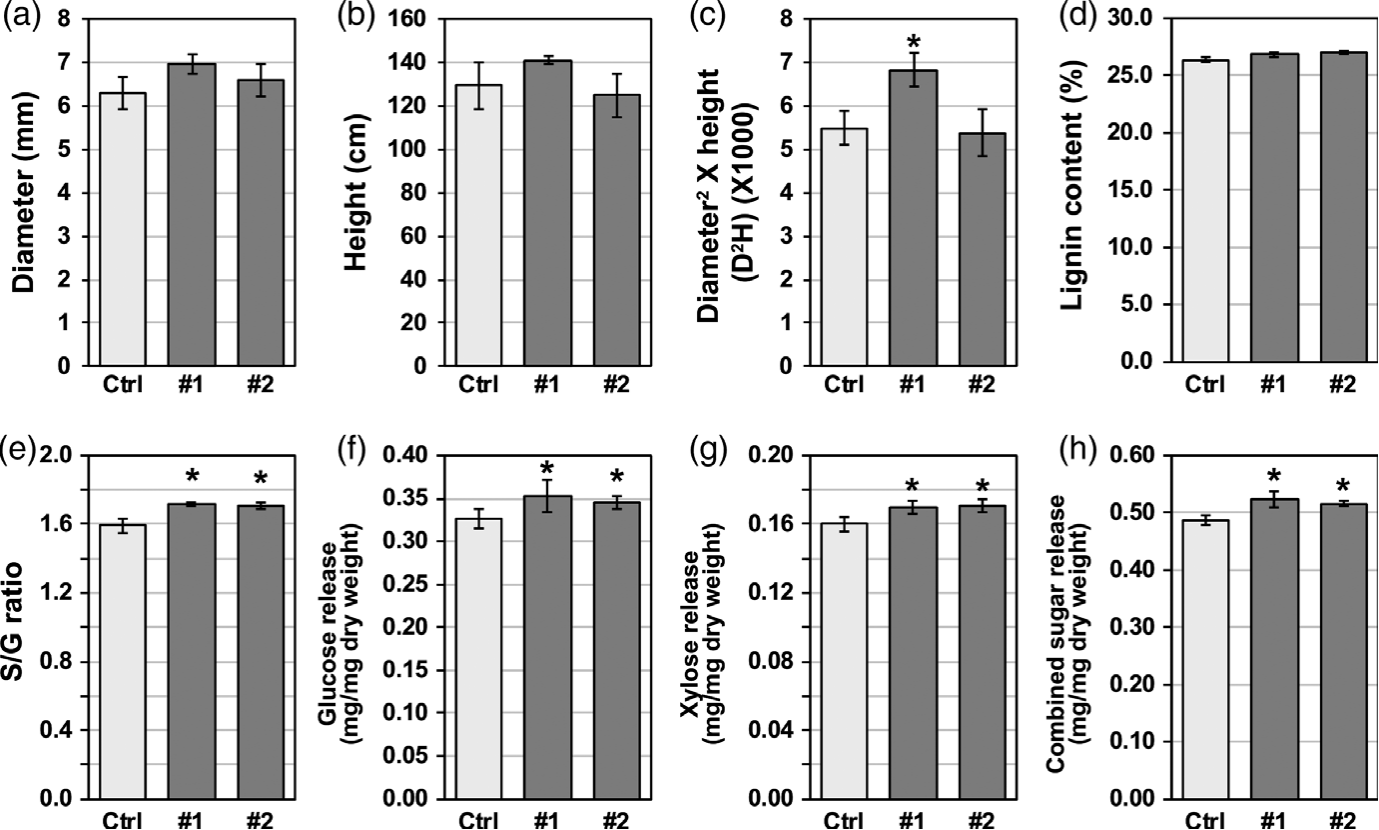
Growth status and cell wall characteristics of *Populus* transgenic plants overexpressing *PdPFD2.2*. (a–c) Growth status of *Populus* transgenic plants overexpressing *PdPFD2.2*. The diameter of basal stem (a) and height (b) were compared between transgenic lines and the empty vector control line (Ctrl). The above‐ground biomass (c) of transgenic poplar samples was estimated using the formula diameter^2^ × height (D^2^H). Lignin content (d) and syringyl/guaiacyl (S/G) lignin ratio (e) in *PdPFD2.2* overexpression lines were compared to those in the Ctrl plants. Glucose (f) and xylose (g) release assay and the combined sugar release with glucose and xylose (h) of *PdPFD2.2* overexpression lines were compared with Ctrl plants. *Significant compared to the control, *P*‐value < 0.05.

Analysis of transgenic lines showed no significant difference in basal stem diameter or plant height compared to empty vector control plants (Figure 3a, b). When both longitudinal and lateral growth rates were combined, line #1 showed a significant increase in above‐ground biomass as measured by diameter^2^ × height (D^2^H) (Figure 3c). Based on molecular beam mass spectrometry (MBMS) measurements, lignin content in the *Populus* transgenic lines overexpressing *PdPFD2.2* was not significantly different from that in the control plants (Figure 3d). However, the lignin S/G ratio was increased by 7.9% and 7.3% in the two *PdPFD2.2* overexpression lines (Figure 3e). In addition, we analysed the cellulose and hemicellulose contents in *PdPFD2.2* overexpression lines. There were no significant differences in cellulose and hemicellulose contents between the control and two overexpression lines (Figure S5a, b). To explore if *PdPFD2.2* overexpression affected cellulose crystallinity, we analysed the cellulose crystallinity index (CrI), the number‐average degree of polymerization (DPn) of cellulose, the weight‐average degree of polymerization (DPw) of cellulose and the polydispersity index (PDI) of cellulose. No significant differences in each of these cellulose parameters were found between control and *PdPFD2.2* overexpression lines (Figure S6), indicating that *PdPFD2.2* may not be associated with cellulose biosynthesis or cellulose crystallinity in *Populus*.

### Saccharification efficiency of *Populus* transgenic plants overexpressing *PdPFD2.2*

To assess the sugar release performance of the *PdPFD2.2* overexpression lines, glucose and xylose release during the enzymatic hydrolysis was compared between transgenic and control plants. As shown in Figure 3f‐h, both glucose and xylose release from the two *PdPFD2.2* overexpression lines was greater than the control plants. The total released sugar from the two lines was increased by 7.6% and 6.1%. In order to rule out the possible effects caused by changes in cellulose and hemicellulose contents, we determined the glucose and xylose release relative to cellulose and hemicellulose contents, respectively, and notably, the *PdPFD2.2* transgenic lines still showed high glucose and xylose release (Figure S5c, d). It should be noted that although there is a relatively large difference (~three‐fold) in the *PdPFD2.2* transcript level in the two overexpression lines (Figure S4), these two transgenic lines displayed similar phenotypes (Figure 3), implying higher *PdPFD2.2* expression may not necessarily result in larger phenotypic effects; presumably, it may have reached the dosage threshold for affecting phenotypes.

### Transcriptome analysis of *Populus* transgenic plants overexpressing *PdPFD2.2*

**Figure 4:**
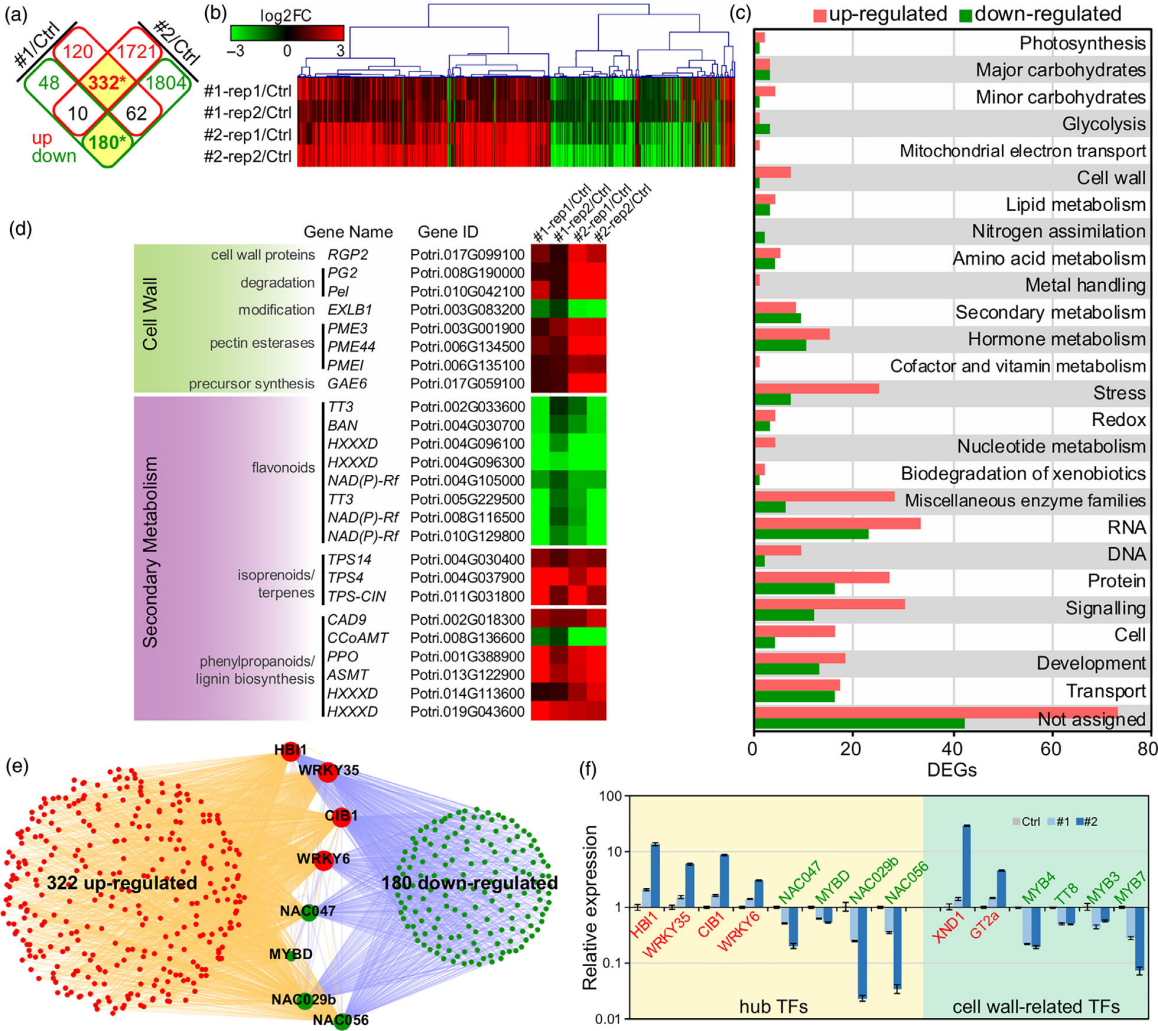
Transcriptomic changes in *Populus* transgenic plants overexpressing *PdPFD2.2*. (a) The differentially expressed genes (DEGs) overlapped in the two *PdPFD2.2* overexpression lines (#1 and #2) compared to controls. Red and green rectangles indicate up‐ and down‐DEGs, respectively. (b) Hierarchical clustering of overlapped DEGs in two lines (core‐DEGs from a). The fold changes (FC) of expression were log2 transformed; red and green represent up‐ and down‐regulation, respectively. (c) Functional classification of DEGs using MapMan. (d) Expression of genes involved in cell wall and secondary metabolism. (e) Eight hub transcription factors (TFs) were identified based on the TF binding sites (TFBS) in the promoter of core‐DEGs. (f) qRT‐PCR validation of expression of hub TFs and known cell wall‐related TFs in *PdPFD2.2* transgenic poplars. *y*‐axis is log10 transformed fold change compared to control plants.

To explore how overexpressing *PdPFD2.2* may affect cell wall‐related phenotypes in poplar, we conducted transcriptomic analysis using RNA‐Seq. For each transgenic line and control plants, library construction and RNA‐Seq analyses were performed using two biological replicates. The correlation (*R* ^2^) between replicates were 0.902, 0.936 and 0.885 in control, line #1 and line #2, respectively (Figure S7), suggesting high repeat accuracy between biological replicates. In the two transgenic lines, a total of 752 (514 up and 238 down) and 4109 (2063 up and 2046 down) differentially expressed genes (DEGs) were identified in line #1 and #2, respectively. Among them, 332 up‐ and 180 down‐DEGs overlapped in the two lines and were defined as core‐DEGs (Figure 4a). The hierarchical clustering of core‐DEGs indicates that the expression patterns of core‐DEGs were consistent among transgenic lines and replicates, and the fold changes of DEGs were higher in #2 than #1 (Figure 4b), which is consistent with the *PdPFD2.2* expression level in the two transgenic lines (Figure S4). We then used the MapMan functional classification system to classify these core‐DEGs. For cell wall‐related MapMan bins, a total of 23 up‐ and 17 down‐DEGs belong to cell wall, secondary metabolism, glycolysis or major/minor carbohydrates. In addition, 43 (27 up and 16 down), 32 (25 up and 7 down) and 31 (18 up and 13 down) DEGs were classified as related to protein, stress and development, respectively (Figure 4c and Table S1). We examined the gene expression patterns in metabolism and found that genes related to cell wall precursor synthesis, including pectin esterase, pectate lyases and polygalacturonases, were up‐regulated in *PdPFD2.2* overexpression lines. In secondary metabolism, genes associated with terpenes and phenylpropanoids biosynthesis were up‐regulated, whereas genes associated with flavonoids were down‐regulated (Figure 4d).

To seek potential hub regulatory transcription factors (TFs) that play key roles in *PdPFD2.2* transgenic poplars, we analysed the TF binding sites (TFBS) in the promoter regions of the 322 up and 180 down core‐DEGs (Table S2). After being ranked, eight TFs with potential to bind to the promoter of most core‐DEGs were identified as hub TFs, which include one *HB1*, one *CIB*, two *WRKYs* (*WRKY6* and *WRKY35*), one *MYB‐like* (*MYBD*) and three *NACs* (*NAC029b*,* NAC047* and *NAC056*) (Figure 4e and Table S3). Furthermore, we selected 14 TFs, including eight hub TFs and six known TFs related with cell wall biogenesis (*XND1*,* GT2a*,* MYB4*,* TT8*,* MYB3* and *MYB7*), to validate their up‐ or down‐expression using qRT‐PCR. As shown in Figure 4f, six TFs were up‐regulated and eight TFs were down‐regulated in the two transgenic lines, which are consistent with the results from RNA‐Seq. It is plausible that these TFs may act downstream of PdPDF2.2 to regulate those cell wall‐related phenotypical changes observed in the *Populus* transgenic plants overexpressing *PdPFD2.2*.

### Metabolite profiles of *Populus* transgenic plants overexpressing *PdPFD2.2*

**Figure 5:**
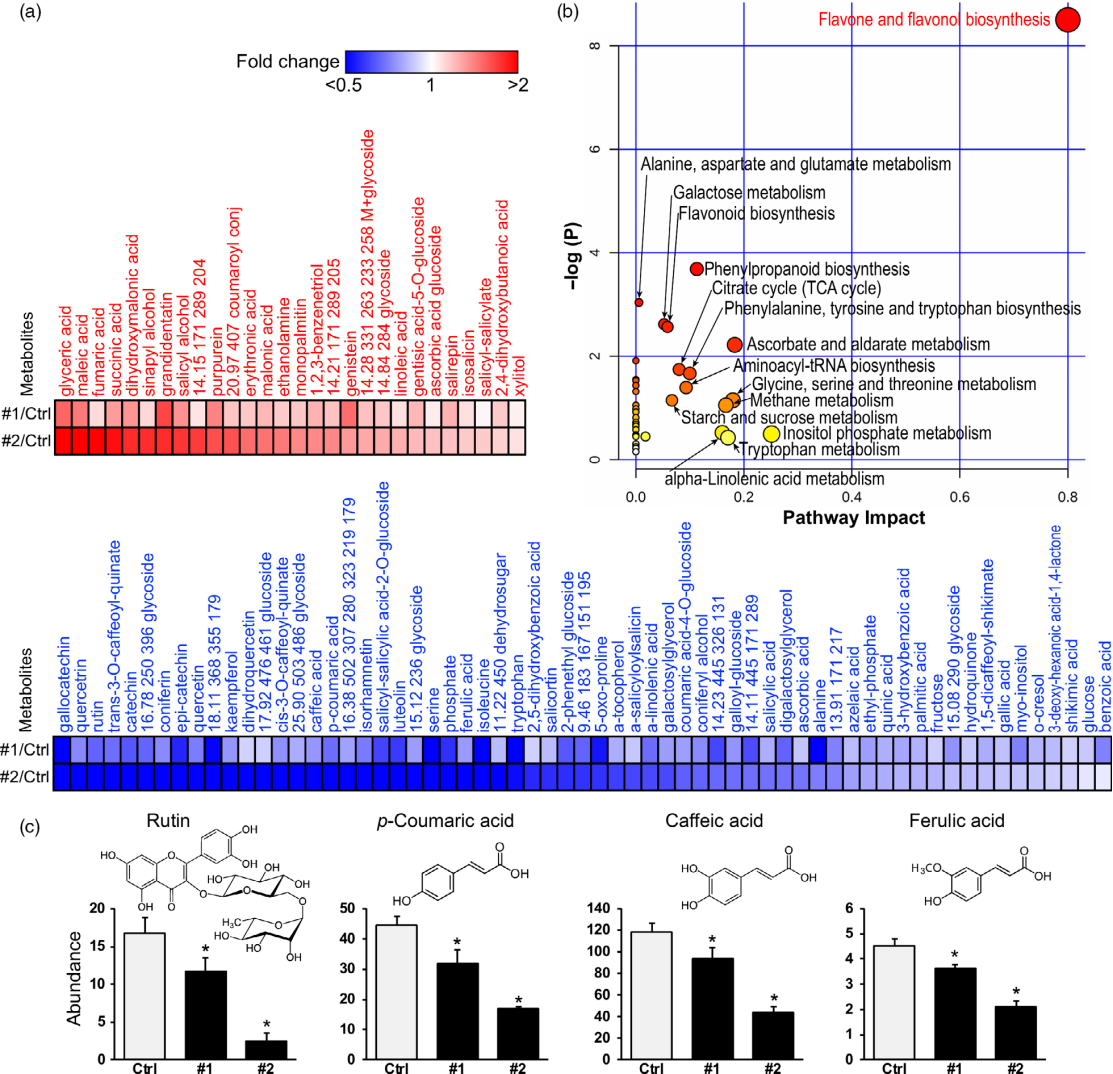
Metabolomic changes of *Populus* transgenic plants overexpressing *PdPFD2.2*. (a) Metabolite profile in mature leaf of two *PdPFD2.2* overexpression lines (#1 and #2) compared to control plants. (b) Pathway analysis of differential metabolites between control (Ctrl) and *Populus* transgenic plants overexpressing *PdPFD2.2*. Global metabolic alterations of the most relevant pathways induced by *PdPFD2.2* were revealed using the MetaboAnalyst. Small *P‐*value and large pathway impact factor indicate that the pathway is greatly influenced. (c) Abundance of rutin, *p*‐coumaric acid (pCA), caffeic acid and ferulic acid (FA) in flavone and flavonol biosynthesis pathway in Ctrl and *PdPFD2.2* overexpression lines. The graphs depict the abundance and chemical structure of these metabolites.

To further examine the function of *PdPFD2.2* at the metabolomic level, we analysed the metabolite profiles of *PdPFD2.2* transgenic lines. Overexpression *PdPFD2.2* had greatly altered the leaf metabolite profiles relative to control plants (Figure 5a). Among a total of 165 identified metabolites by GC‐MS, 91 metabolites were changed, including 28 up‐ and 63 down‐regulated metabolites in *PtPFD2.2* transgenic lines (Figure 5a). Based on the metabolic pathway analysis, the most enriched pathway from the 91 metabolites was ‘flavone and flavonol biosynthesis’, followed by ‘phenylpropanoid biosynthesis’, ‘alanine, asparate and glutamate metabolism’, ‘galactose metabolism’ and ‘flavonoid biosynthesis’ (Figure 5b). The flavone and flavonol biosynthesis pathway include the synthesis of numerous flavonoid metabolites, and it was this class of metabolites that was most down‐regulated in the two transgenic lines (Figure 5c), including rutin [Figure 5c; a glycoside of the flavonol quercetin that is conjugated to the disaccharide rutinose (α‐L‐rhamnopyranosyl‐(1→6)‐β‐D‐glucopyranose)], luteolin, isorhamnetin, dihydroquercetin (taxifolin), kaempferol, catechin, epi‐catechin, quercetin and gallocatechin (Figure 5a). In contrast, one flavonoid that was up‐regulated was genistein (GNT) (Figure 5a). Examples of phenylpropanoid metabolites that were significantly reduced included the upstream hydroxycinnamate precursors of lignin biosynthesis, *p*‐coumaric acid (*p*CA), caffeic acid and ferulic acid (FA), and the downstream products, coniferyl alcohol (G‐monolignol), coniferin and its glucoside storage product (Figure 5a, c). Notably, sinapyl alcohol (S‐monolignol) was up‐regulated (Figure 5a), which is consistent with increased S/G ratios observed in the *PdPFD2.2* transgenic plants (Figure 3e).

Whereas most flavonoids declined, secondary metabolites of the higher‐order salicylate [salicylic acid (SA), its esters and glucoside conjugates] pathway were both up and down‐regulated. For example, grandidentatin and purpurein were accumulated in transgenic lines, as were several simple salicylate‐related metabolites and simple conjugates, including salicyl alcohol, gentisic acid 5‐O‐glucoside, salirepin, isosalicin and salicyl‐salicylate (Figure 5a). Notably, several of the larger, more complex higher‐order salicylate metabolites were down‐regulated in transgenic lines, including salicortin and salicyl‐salicyloyl‐2‐*O*‐glucoside, as well as SA (Figure 5a). Taken together, these results further validate the role of PFD2.2 in metabolite responses and are consistent with changes in cell wall properties and the transcriptome of *Populus* transgenic plants overexpressing *PdPFD2.2*.

## Discussion

As a group II chaperonin, PFD plays important roles in newly synthesized protein folding and protection (Cao, 2016). Several studies in different species have also indicated that PFD is involved in cytoskeleton organization (Geissler *et al*., 1998; Gu *et al*., 2008; Lundin *et al*., 2008) and transcriptional regulation (Locascio *et al*., 2013; Millan‐Zambrano and Chavez, 2014; Millan‐Zambrano *et al*., 2013). However, its function in plants is largely unknown. In this study, we provide evidence that a *Prefoldin β* subunit gene in *Populus*,* PtPFD2.2*, has a role in modifying cell wall properties and that *PtPFD2.2* can be used to reduce biomass recalcitrance in the bioenergy crop *Populus*.

The co‐expression network and posttranslation modification indicate that many proteolysis‐related proteins are co‐expressed with *PtPFD2.2* (Figure S1). The occurrence of ubiquitination and sumoylation sites (Figure S3) is consistent with *PtPFD2.2* functioning as a chaperonin. Cell wall‐related genes were co‐expressed with *PtPFD2.2* (Figure 1), and *PtPFD2.2* was highly expressed in cambium (Figure 1). These imply that *PtPFD2.2* may have a role in cell wall‐related processes. In *Arabidopsis*,* PFD* is involved in cytoskeleton‐related processes, including microtubule dynamic and organization. The *pfd6‐1* mutant has reduced tubulin proteins abundance and exhibits defects in cell division and microtubule dynamicity (Gu *et al*., 2008). The components of cytoskeleton are tightly connected with cambium cell division and secondary vascular differentiation in woody perennial plants. For example, in dividing cambial cells, myosin and callose are localized at the cell plate, while actin microfilaments (MFs) are alongside the cell plate; both tubulin and actin are associated with the phragmoplast (Chaffey and Barlow, 2002). MFs were rare in ray cambial cells; but were abundant and axially arranged in their derivatives once cell elongation had begun. Microtubules were randomly oriented in ray and fusiform cells of the cambial zone (Chaffey *et al*., 2002). Thus, our findings suggest that the altered cell wall properties in the *PdPFD2.2* transgenic poplars might be due to its action in cytoskeleton‐related processes.

In *Arabidopsis*, PFD regulation of cortical microtubule organization depends on its physical interaction with DELLA proteins, which are nuclear proteins that mediate transcriptional regulation of cell expansion and other developmental processes by GAs (Cheng *et al*., 2004; de Lucas *et al*., 2008; Ubeda‐Tomas *et al*., 2008). In rice, a DELLA protein SLR1 can interact with a NAC TF in the top layer of secondary wall formation to regulate cellulose biosynthesis (Huang *et al*., 2015). In the present study, we found that PFD2.2 physically interacts with a DELLA protein RGA1_8g in *Populus*, but not with the other tested DELLA protein, RGA1_10g (Figure 2). The differences in protein–protein interaction of the two DELLA proteins with PFD2.2 might be caused by the protein structure difference of RGA1_8g and RGA1_10g (Figure 2). The nuclear localization of PFD2.2 is likely guided by RGA1_8g. However, the cellulose content was not significantly affected in the *Populus* transgenic plants overexpressing *PdPFD2.2*, suggesting that the interaction of PFD2.2‐DELLA is not directly involved in cellulose biosynthesis in *Populus*.

As a key player in phytohormone cross‐talk, DELLA is also involved in other hormone signalling pathways. For example, DELLA‐like1 is co‐localized with endogenous biologically active GAs in expanding xylem cells in poplar (Israelsson *et al*., 2005) and in a feedback response to increased GA and IAA levels (Bjorklund *et al*., 2007). DELLA can also alter the relative strength of defense hormones SA and JA. In *Arabidopsis*, DELLA has different effects on SA and JA. Navarro *et al*. (2008) reported DELLA promoted JA perception and/or signalling and antagonized SA to modulate immunity. Unlike in *Arabidopsis*, a rice DELLA protein, Slender Rice 1 (SLR1), acts as a positive regulator of hemibiotroph resistance by integrating and amplifying SA‐ and JA‐dependent defense signalling (De Vleesschauwer *et al*., 2016). In the present study, overexpression of *PdPFD2.2* altered the SA‐related metabolites (e.g. salicyl‐salicyloyl‐2‐*O*‐glucoside, salicortin, salicyl alcohol and salicylate) in transgenic poplar (Figure 5a), which provides clues for the connection between *PdPFD2.2* and the SA signalling pathway. Furthermore, WRKYs belong to a large family of TFs and are known as key regulators responding to pathogen attack and to the endogenous SA (Eulgem and Somssich, 2007). In addition, WRKY TFs are involved in the activation of SA biosynthesis. For example, WRKY28 and WRKY46 directly activate the expression of SA biosynthesis genes *ICS1* and *PBS3* (van Verk *et al*., 2011). In the present study, two WRKYs (WRKY6 and WRKY35) were identified as potential downstream regulators of *PdPFD2.2* in RNA‐Seq analysis (Figure 4e), implying that *PdPFD2.2* may also potentially be involved in defense‐related processes, but this has not been experimentally tested.

Among the eight hub TFs, three *NAC* (*NAC029b*,* NAC047* and *NAC056*) and one *MYB* (*MYBD*) were down‐regulated (Figure 4e). In contrast, other known TFs involved in cell wall‐related processes, such as *XND1*,* MYB3*,* MYB4*,* MYB7* and *TT8*, were also affected in *Populus* transgenic plants overexpressing *PdPFD2.2* (Figure 4f). *XND1* is a negative regulator of lignocellulose synthesis and programmed cell death in xylem (Zhao *et al*., 2008), and it was up‐regulated by *PdPFD2.2* overexpression (Figure 4f). Among the four down‐regulated TFs (*MYB3*,* MYB4*,* MYB7* and *TT8*), *MYB3* is a repressor of phenylpropanoid biosynthesis, *MYB4* is a repressor of lignin biosynthesis, and *MYB7* and *TT8* work as repressor and activator in flavonol biosynthesis, respectively (Zhang *et al*., 2018). The changes of these key secondary metabolic TFs by *PdPFD2.2* is likely the primary mechanism driving the flavonoid responses and the hydroxycinnamate and monolignol responses of the lignin pathway.

Generally, cell wall recalcitrance is gauged by biomass enzymatic hydrolyzability (McCann and Carpita, 2015). Among various components in cell walls, lignin is one of the most significant recalcitrance contributors (Pu *et al*., 2011). In biomass, cell wall recalcitrance was related to lignin content, structure, subunit composition and linkages with polysaccharides. The relative abundance of H‐, S‐ and G‐units of lignin have been proved to be associated with biomass recalcitrance. Biomass recalcitrance is a multi‐scale, multi‐factor property that is influenced by a variety of factors such as lignin content, lignin structural features, cellulose degree of polymerization and crystallinity, hemicellulose content/structures and accessibility (Meng *et al*., 2017). In this study, although overexpression of *PdPFD2.2* slightly increased the lignin content in the transgenic lines, the increase was not statistically significant when compared to the control plant. On the other hand, S/G ratio was significantly increased in the *PdPFD2.2* transgenic plants, compared to the control plant. Because among all the examined cell wall‐related parameters (Figures 3, S5, S6), including the content of lignin, cellulose or hemicellulose, cellulose crystallinity index and degree of cellulose polymerization, lignin S/G ratio was the only changed parameter in the *PdPFD2.2* transgenic plants, we deduced that the increased sugar release observed in the *PdPFD2.2* transgenic plants (Figure 3) is likely due to the increased lignin S/G ratio.

It has been reported that the influence of S/G ratio on the hydrolyzability of biomass varies on a number of factors, such as plant species, lignin content, pretreatment method, enzyme types, as well as natural and transgenic lines (Li *et al*., 2016b). For example, Chen reported that S/G ratio alone did not correlate well with the sugar release in transgenic alfalfa lines (Chen and Dixon, 2007). Lignin S/G ratio was also reported to have negative effect on biomass saccharification in *Miscanthus* after alkaline pretreatment (Li *et al*., 2014; Xu *et al*., 2012), which was partially attributed to the effective co‐extraction of lignin–hemicellulose complexes in G‐rich lignin samples under the alkaline pretreatment conditions. For poplar wood, saccharification was positively correlated with S/G ratio in poplar biomass after hot water pretreatment (Studer *et al*., 2011) and steam explosion pretreatment (Mansfield *et al*., 2012). The positive effect of high S/G ratio on saccharification has been related to the S‐lignin structural features: (i) higher level of labile β‐O‐4 linkages in S‐lignin which are readily cleavable during pretreatment and (ii) relatively higher occurrence of β‐β bonds in S‐lignin leading to lower molecular weight which could facilitate lignin migration and removal during pretreatment. It was also reported that the G unit of lignin could form a more cross‐linked lignin structure generating a larger physical barrier against the substrate accessibility (Yu *et al*., 2014). These results further suggest the complexity of the influence of S/G ratio on saccharification of biomass and the difficulty in comparison between various biomass species and pretreatments. In this study, the sugar release performance of poplar was measured after hot water pretreatment by using a method similar to the one used by Studer *et al*. (2011), further supporting that higher lignin S/G ratio improves sugar release under this condition. Furthermore, in a recent study (Alam *et al*., 2019), biomass porosity and cellulose accessibility were proposed as the finalized determinant on biomass enzymatic hydrolysis. It would be worthwhile to investigate whether the increased S/G ratio in the *PdPFD2.2* transgenic plants affects the biomass porosity and the cellulose surface accessibility after chemical pretreatments for co‐extraction of lignin and hemicellulose in future studies.

Recalcitrance is also associated with the hydroxycinnamates in lignin, which include *p*CA, FA, caffeic acid, etc. (Li *et al*., 2016b). *p*CAs are usually present as esters pendantly linked at the γ‐position of S‐lignin and acylated to polysaccharides (Petrik *et al*., 2014). In maize, the cell wall enzymatic digestibility is negatively correlated with the esterified *p*CA and lignin content (Zhang *et al*., 2011). In contrast, FA mediates the cross‐link reaction of polysaccharides‐lignin and polysaccharides‐polysaccharides (Azarpira *et al*., 2011) and reduced FA‐mediated cross‐linking improved digestibility (Jung and Phillips, 2010). In *Populus* transgenic plants overexpressing *PdPFD2.2*, the contents of *p*CA, FA and caffeic acid were significantly decreased (Figure 5), which is consistent with its reduced recalcitrance.

In summary, overexpression of *PdPFD2.2* enhances lignin S/G ratio and sugar release in transgenic poplar. Therefore, *PdPFD2.2* plays a role in overcoming plant cell wall recalcitrance, which provides a path to reduce recalcitrance via modifying cell wall composition. As such, *PdPFD2.2* can be a potential target for genetic engineering to improve biofuel conversion and reduce the cost of lignocellulosic biofuel production.

## Experimental procedures

### Plant materials

The full‐length open‐reading frame of *PdPFD2.2* was amplified from *Populus deltoides* ‘WV94’. The complementary DNA (cDNA) was cloned into the pAGW560 binary vector in which the expression of *PdPFD2.2* was driven by the *Ubiquitin 3* promoter. Agrobacterium‐mediated transformation into *P. deltoides* ‘WV94’ was conducted at ArborGen Inc. (Ridgeville, SC) (Biswal *et al*., 2015). Then transgenic plants were transferred and grown in the greenhouse at Oak Ridge National Laboratory (Oak Ridge, TN) at 25 **°**C and 16 h/8 h photoperiod. To estimate stem cylinder volume, plant height and stem base diameter were measured in ~6‐month‐old plants. We measured primary stem length from stem base to shoot tip for plant height and measured the diameter of stem base.

### Bioinformatics analysis

To identify PFD2 protein in *Populus*, the full‐length amino acid sequence of *Arabidopsis* PFD2 (AT3G22840) was subjected to Blastp search integrated in the Phytozome (https://phytozome.jgi.doe.gov/pz/portal.html). Each identified PtPFD2 homolog was then used as a new query to search for the PFD2 protein in *Salix purpurea*,* Eucalyptus grandis*,* Vitis* vinifer, *Medicago truncatula*,* Glycin max*,* Oryza sativa*,* Zea mays*,* Brachypodium distachyon*,* Sorghum bicolor* and moss (*Physcomitrella patens*) genomes. The full‐length amino acid sequences with *E‐*value < 1E‐10 were selected and subjected to the Pfam database (http://pfam.xfam.org/) to validate the presence of the Prefoldin_2 domain PF01920.

To conduct a phylogenetic analysis, a maximum likelihood (ML) tree was constructed with full‐length amino acid sequences of collected PFD2 proteins. Multiple sequence alignment of PFD2 proteins were performed using the Clustal X2.1 (Larkin *et al*., 2007). A phylogenetic tree was constructed using maximum likelihood (ML) method by MEGA7 with 1000 bootstrap replicates (Kumar *et al*., 2016).

Normalized FPKM values of the *PtPFD2* genes from various tissues and organs were obtained from the *Populus* Atlas database integrated in Phytozome (https://phytozome.jgi.doe.gov/pz/portal.html), which includes 24 samples from bud (predormant bud I, predormant bud II, early dormant bud, late dormant bud and fully opened bud), leaf (young, immature and first fully expanded leaf), stem (inode, node, stem_ammonia, stem_nitrate and stem_urea), root (root, root tip, root_ammonia, root_nitrate and root_urea), female catkin (BESC423.ZL 7 female early, BESC842.ZI 22 female late and BESC443.ZG 43 female receptive) and male catkin (GW9592.ZK 10 male early, GW9840.ZE 30 male early and GW9911.ZK 51 male mid). For gene expression during wood formation, a nm‐scale high‐resolution gene expression database AspWood (http://aspwood.popgenie.org/aspwood-v3.0/) was used, which includes 25 samples from phloem, cambium and expanding and maturing xylem. A co‐expression network of *PtPFD2.2* was created according to the method reported by Li *et al*. (2016a) using data obtained from the co‐expressed biological processes database for *P. trichocarpa* (http://webs2.kazusa.or.jp/kagiana/cop0911/). Cytoscape (Smoot *et al*., 2011) was used to visualize the resulting network.

Three‐dimensional structure prediction was performed by means of the I‐TASSER (iterative threading assembly refinement) suite (Yang *et al*., 2015).

### Subcellular localization

For the analysis of protein subcellular localization, PFD2.2, RGA1_8g and RGA1_10g were cloned into the YFP fusion vector and expressed in the *Populus* leaf mesophyll protoplasts (Xie *et al*., 2018). Specifically, 8 μg of YFP‐PFD2.2, YFP‐RGA1_8g and YFP‐RGA1_10g constructs were co‐transfected with 2 μg of VirD2NLS‐mCherry construct into 100 μL of protoplasts, respectively. After a 12‐h incubation under weak light, YFP and mCherry fluorescence were examined and photographed. Images were collected on a Zeiss LSM 710 confocal microscope and were processed using the Zeiss ZEN software package.

### Bimolecular fluorescence complementation (BiFC) assay

For BiFC, CDS sequence of *PdPFD2.2* was cloned into the pSAT1‐cCFP‐C vector (ABRC# CD3‐1068). RGA1_8g or RGA1_10g was cloned into the pSAT1‐nVenus‐C vector (ABRC# CD3‐1076). Then, each of the expression cassettes of these three constructs were cloned into the transient expression vector pUC119‐RCS (ABRC# CD3‐1747) by AscI. To perform BiFC, 8 μg of cCFP construct, 8 μg of nVenus construct and 4 μg of nuclear marker VirD2NLS‐mCherry construct were co‐transfected into 200 μL of protoplasts. After a 12‐h incubation under weak light, YFP and mCherry fluorescence were examined and photographed. Images were collected on a Zeiss LSM 710 confocal microscope and were processed using the Zeiss ZEN software package.

### Chemical composition analysis

Four milligrams of dried, ground [40 mesh] stem biomass was placed into a pyrolysis molecular beam mass spectrometry chamber, and then, using 17 eV electron impact ionization, mass spectral data were acquired on a Merlin Automation data system version 3.0 from 30 to 450 m/z (Sykes *et al*., 2009). Lignin estimates were determined as described previously (Sykes *et al*., 2009). S/G ratios were determined by summing the area under the peaks attributed to syringyl moieties (i.e. m/z 154, 167, 168, 182, 194, 208 and 210), and dividing this area by the area under the peaks attributed to guaiacyl moieties (i.e. m/z 124, 137, 138, 150, 164 and 178). Cellulose and hemicellulose were analysed according to Methods S1.

### Gel permeation chromatographic (GPC) analysis

The weight‐average molecular weight (M_w_) and number‐average molecular weight (M_n_) of cellulose were measured by GPC after tricarbanilation (Li *et al*., 2017). Briefly, the α‐cellulose was derivatized with phenyl isocyanate in an anhydrous pyridine system prior to GPC analysis. Size‐exclusion separation was performed on an Agilent 1200 HPLC system (Agilent Technologies, Inc, Santa Clara, CA) equipped with Waters Styragel columns (HR1, HR2 and HR6; Waters Corporation, Milford, MA). Number‐average degree of polymerization (DP_n_) and weight‐average degree of polymerization (DP_w_) of cellulose were obtained by dividing M_n_ and M_w_, respectively, by 519 g/mol, the molecular weight of the tricarbanilated cellulose repeating unit. The results were reported as the average value of duplicate measurements.

### Cellulose crystallinity analysis by solid‐state NMR

Cellulose crystallinity was measured with cross‐polarization magic angle spinning (CP/MAS) solid‐state NMR according to published procedure (Li *et al*., 2017). Cellulose for solid‐sate NMR analysis was isolated from the holocellulose (150 mg) by hydrolysis at 100 **°**C with HCl (8 mL of 2.5 m) for 2 h. The isolated cellulose was retained at 30%–50% moisture. The NMR samples were packed into 4‐mm cylindrical Zirconia MAS rotors. CP/MAS NMR analysis of cellulose was carried out on a Bruker Avance‐400 spectrometer operating at frequencies of 100.59 MHz for ^13^C in a Bruker double‐resonance MAS probe head at spinning speeds of 8 kHz. CP/MAS experiments utilized a 5 ms (90 **°**) proton pulse, 1.5 ms contact pulse, 4 s recycle delay and 2048 scans. The cellulose crystallinity index (CrI) was determined from the areas of the crystalline and amorphous C‐4 signals using the following formula:

$$\frac{A_{86-92\mathrm{ppm}}}{A_{86-92\mathrm{ppm}}+A_{79-86\mathrm{ppm}}}$$

### Saccharification assay

Dried and Wiley‐milled (40 mesh) stems of the *Populus* control and transgenic plants were used for saccharification assays according to the method described by Bryan *et al*. (2016). Detailed method is shown in Methods S2.

### RNA‐Seq analysis

Fully expanded leaves were ground in liquid nitrogen, and total RNA was extracted using a Spectrum Total Plant RNA extraction kit (Sigma‐Aldrich, St. Louis, USA) with the on‐column RNase‐free DNase I treatment to remove the residual genomic DNA. RNA quality and quantity were determined using a Nanodrop Spectrophotometer (Thermo Fisher Scientific, Hudson, NH). RNA‐Seq libraries were generated and quantified using qPCR. Sequencing was performed on an Illumina HiSeq 2500 (150mer paired‐end sequencing). Reads mapping and differential expression analysis were performed according to Methods S3. For functional analysis, genes were classified using MapMan (Thimm *et al*., 2004). GO enrichment was performed using agriGO (Tian *et al*., 2017). For the promoter analysis, the transcription factor binding sites were identified using PlantPAN (Chow *et al*., 2016).

### Metabolomic analysis by gas chromatography‐mass spectrometry (GC‐MS)

Leaves (LPI 6) of ~6‐month‐old *PdPFD2.2* transgenic lines (#1 and #2) and control plants (*n* = 9; 3 plants from each line) growing in the greenhouse were fast frozen in liquid nitrogen and stored at −80 **°**C. Metabolomic analysis was performed according to Methods S4.

### qRT‐PCR analysis

One microgram of total RNA was used to generate cDNA by means of the Rite aid reverse transcriptase following manufacturer's instruction (Thermo Fisher Scientific, Hudson, NH). Gene‐specific primers were designed using Primer3 software (http://frodo.wi.mit.edu/primer3/input.htm) with annealing temperature of 58–60 **°**C and amplicon size of 150–250 bp. qRT‐PCR was performed using Maxima SYBR Green/ROX qPCR master mix (Thermo Fisher Scientific) according to the manufacturer's instructions. The relative gene expression was calculated by 2^−ΔΔCt^ method (Livak and Schmittgen, 2001) using *PtUBQ10b* (*Potri.001G263000*) as internal control. All the experiments were repeated at least three times with similar results. The primers used in this study are listed in Table S4.

### Statistical analysis

Statistical analysis to determine statistical significance was performed by Student's *t*‐tests of paired samples. The asterisk in each figure indicates significant difference compared to control samples (*P *≤* *0.05).

## Conflict of interest

The authors declare no conflict of interest.

## Author contributions

JZ, WM and JGC designed this study. JZ performed experiments, conducted dada analysis and wrote the manuscript. MX performed subcellular localization analysis. ML and JD performed chemical compositional analysis. WR, KAW and CMC generated *Populus* transgenic lines. VS, EAL, KB and JS generated RNA‐Seq data. ACB, SSJ and LEG measured biomass production. NLE and TJT generated and analysed metabolomics data. XY designed the construct for *Populus* transformation. GAT, WM and JGC conceived the study, coordinated research and contributed to experimental design and data interpretation. JZ, YP, TJT, GAT and JGC revised the manuscript. All authors read and approved the final manuscript.

## Supporting information


**Methods S1** Cellulose and hemicellulose analysis.
**Methods S2** Saccharification assay.
**Methods S3** RNA‐Seq analysis.
**Methods S4** Metabolomic analysis by gas chromatography‐mass spectrometry (GC‐MS).
**Figure S1** GO enrichment of genes co‐expressed with *PtPFD2.2*.
**Figure S2** *cis*‐acting elements in the promoter region of *PtPFD2.2*.
**Figure S3** Protein structure of PtPFD2.2. (a) Secondary protein structure of PtPFD2.2 with potential post‐translation modification sites. Yellow, red and blue pins represent phosphorylation, sumoylation and ubiquitination sites, respectively. Conserved Prefoldin_2 motifs identified from pfam database are shown in the blue box (13–118 aa). (b) Protein 3D structure of PtPFD2.2.
**Figure S4** Expression of *PdPFD2.2* in control (Ctrl) and two overexpression lines through qRT‐PCR.
**Figure S5** Cellulose and hemicellulose contents and sugar release based on cellulose or hemicellulose in *PdPFD2.2* overexpression lines. (a) Cellulose content. (b) Hemicellulose content. (c) Glucose release based on the cellulose content. (d) Xylose release based on the hemicellulose content.
**Figure S6** Cellulose crystallinity index (CrI) and degree of polymerization (DP) of cellulose in *Populus* transgenic lines overexpressing *PdPFD2.2*. (a) CrI measurement using solid‐state NMR. (b) The number‐average degree of cellulose polymerization (DPn). (c) The weight‐average degree of cellulose polymerization (DPw). (d) The polydispersity index (PDI) of cellulose.
**Figure S7** Correlation of biological replicates in RNA‐Seq analysis.

**Table S1** Functional classification of core‐DEGs by MapMan
**Table S2** TF list from core‐DEGs in RNA‐Seq of *PdPFD2.2* overexpression lines
**Table S3** Eight hub TFs of the core‐DEGs
**Table S4** qRT‐PCR primers used for this study
