# Supplementary material for: Overexpression of a Prefoldin β subunit gene reduces biomass recalcitrance in the bioenergy crop Populus
Source: Plant Biotechnol J. 2019 Sep 27;18(3):859–71. doi: 10.1111/pbi.13254 (PMC7004918; doi:10.1111/pbi.13254)

Supporting Information

Overexpression of a *Prefoldin β* subunit gene reduces biomass recalcitrance in the bioenergy crop *Populus*

Jin Zhang[1,2], Meng Xie[1,2,3], Mi Li[4], Jinhua Ding[4,5], Yunqiao Pu[1,2], Anthony C. Bryan[1], William Rottmann[6], Kimberly A. Winkeler[6], Cassandra M. Collins[6], Vasanth Singan[7], Erika A. Lindquist[7], Sara S. Jawdy[1,2], Lee E. Gunter[1,2], Nancy L. Engle[1,2], Xiaohan Yang[1,2], Kerrie Barry[7], Timothy J. Tschaplinski[1,2], Jeremy Schmutz[7,8], Gerald A. Tuskan[1,2], Wellington Muchero[1,2,*] and Jin-Gui Chen[1,2,*]

[1] Biosciences Division, Oak Ridge National Laboratory, Oak Ridge, TN 37831, USA
[2] Center for Bioenergy Innovation, Oak Ridge National Laboratory, Oak Ridge, TN 37831, USA
[3] Department of Plant Sciences, University of Tennessee, Knoxville, TN 37996, USA
[4] Chemical & Biomolecular Engineering, University of Tennessee, Knoxville, TN 37996, USA
[5] College of Textiles, Donghua University, Shanghai, 201620, P.R. China
[6] ArborGen Inc., Ridgeville, SC 29472, USA
[7] U.S. Department of Energy Joint Genome Institute, Walnut Creek, CA 94598, USA
[8] HudsonAlpha Institute for Biotechnology, Huntsville, AL 35806, USA

Methods S1. Cellulose and hemicellulose analysis.

For cellulose and hemicellulose isolation, the extractives-free samples were delignified by peracetic acid with 5.00 g loading per g biomass. The solution consistency was adjusted to 5% with deionized (DI) water and the holopulping was conducted at room temperature for 24 h with magnetic stirring. The solid residue, designated as holocellulose, was washed with excessive DI water (18.0 MΩ) and air dried at room temperature for 24 h. A sub-portion of the air-dried holocellulose (100 mg) was consecutively extracted at 25°C with 17.5% (wt/v) NaOH solution (5.00 mL) for 2 h, followed by 8.75% NaOH solution (10.00 mL) for an additional 2 h. The alkaline slurry was then filtered and rinsed with 5 mL of 1% acetic acid leading to a liquid fraction and a solid residue. The solid residue, namely α-cellulose, was washed with an excess of DI water and air dried for the analysis of cellulose DP. The liquid fraction, rich in hemicellulose, was adjusted to pH 6-7 with anhydrous acetic acid. Hemicellulose was then precipitated by adding three volumes of 100% ethanol to the liquid fraction. Hemicellulose was then obtained by centrifugation at 8000 rpm (267π rad/s) for 5 min and freeze dried for 24 h.

Methods S2. Saccharification assay.

Dried and Wiley-milled (40 mesh) stems of the *Populus* control and transgenic plants were used for saccharification assays. In brief, biomass was extracted with α-amylase (Spirizyme Ultra, 0.25%) and α-glucosidase (Liquozyme SC DS, 1.5%) in 0.1 M sodium acetate (24 h, 55°C, pH 5.0) to remove possible starch content; followed by an ethanol (95% v/v) Soxhlet extraction for an additional 24 h. After drying overnight, 5 mg (±0.5 mg) of biomass was weighed in triplicate into one of 96 wells in a solid Hastelloy microtitre plates and 250 µL of water was added. Samples were then sealed with silicone adhesive, Teflon tape. For pretreatment, the samples were reacted at 180°C for 17.5 min, after cooled, 40 µL of buffer-enzyme stock [8% CTec2 (Novozymes, Bagsværd, Denmark) (excess enzyme loading of 70 mg/g biomass) in 1 M sodium citrate buffer] was added. The samples were then gently mixed and left to statically incubate at 50°C for 70 h. After incubation, an aliquot of the saccharified hydrolysate was diluted and tested using megazymes GOPOD (glucose oxidase/peroxidase) and XDH assays (xylose dehydrogenase). Results were calculated using standard curves created from mixtures of glucose and xylose.

Methods S3. RNA-Seq analysis.

Raw fastq file reads were filtered and trimmed using the JGI QC pipeline. Using BBDuk (https://sourceforge.net/projects/bbmap/), raw reads were evaluated for sequence artifacts by kmer matching (kmer=25) allowing 1 mismatch, and detected artifacts were trimmed from the 3' end of the reads. RNA spike-in reads, PhiX reads and reads containing any Ns were removed. Quality trimming was performed using the phred trimming method set at Q6. Following trimming, reads under the length threshold were removed (minimum length 25 bases or 1/3 of the original read length; whichever was longer). Raw reads from each library were aligned to the *P. trichocarpa* reference genome (https://phytozome.jgi.doe.gov/pz/portal.html#) using TopHat2 (Kim et al., 2013). Only reads that mapped uniquely to one locus were counted. FeatureCounts (Liao et al., 2014) was used to generate raw gene counts, which were used to evaluate the level of correlation between biological replicates. DESeq2 (Love et al., 2014) was subsequently used to determine which genes were differentially expressed between pairs of conditions (P value < 0.05).

Methods S4. Metabolomic analysis by gas chromatography-mass spectrometry (GC-MS).

The leaf tissues were ground with liquid nitrogen in a chilled mortar and pestle with ~ 50 mg FW; and were subsequently twice extracted with 2.5 mL 80% ethanol overnight and then combined prior to drying a 1 mL aliquot in a nitrogen stream. Sorbitol was added before extraction as an internal standard to correct for differences in extraction efficiency, subsequent differences in derivatization efficiency, and changes in sample volume during heating. Dried extracts were dissolved in 500 µL of silylation–grade acetonitrile, followed by the addition of 500 µL N-methyl-N-trimethylsilyltrifluoroacetamide (MSTFA) with 1% trimethylchlorosilane (TMCS) (Thermo Scientific, Bellefonte, PA), and samples then heated for 1 h at 70°C to generate trimethylsilyl (TMS) derivatives (Tschaplinski et al., 2012). After 2 days, 1-µL aliquots were injected into an Agilent Technologies Inc. (Santa Clara, CA) 5975C inert XL GC–MS, fitted with an Rtx-5MS with Integra-guard (5% diphenyl/95% dimethyl polysiloxane) 30 m × 250 µm × 0.25 µm film thickness capillary column. The standard quadrupole GC–MS was operated in the electron impact (70 eV) ionization mode, targeting 2.5 full-spectrum (50–650 Da) scans per second, as described previously (Tschaplinski et al., 2012). Metabolite peaks were extracted using a key selected ion, characteristic m/z fragment, rather than the total ion chromatogram, to minimize integrating co-eluting metabolites. The extracted peaks of known metabolites were scaled back up to the total ion current using predetermined scaling factors. Peaks were quantified by area integration and concentrations normalized to the quantity of the internal standard (sorbitol) recovered, amount of sample extracted, derivatized, and injected. A large user-created database (> 2400 spectra) of mass spectral electron impact ionization (EI) fragmentation patterns of TMS-derivatized compounds, as well as the Wiley Registry 10th Edition combined with NIST 2014 mass spectral database, were used to identify the metabolites of interest to be quantified. Unidentified metabolites were denoted by their retention time as well as key mass-to-charge (*m/z*) ratios and partial naming given the typical identity of specific *m/z*.

References:

Kim, D., Pertea, G., Trapnell, C., Pimentel, H., Kelley, R. and Salzberg, S.L. (2013) TopHat2: accurate alignment of transcriptomes in the presence of insertions, deletions and gene fusions. *Genome Biol* **14**, R36.

Liao, Y., Smyth, G.K. and Shi, W. (2014) featureCounts: an efficient general purpose program for assigning sequence reads to genomic features. *Bioinformatics* **30**, 923-930.

Love, M.I., Huber, W. and Anders, S. (2014) Moderated estimation of fold change and dispersion for RNA-seq data with DESeq2. *Genome Biol* **15**, 550.

Tschaplinski, T.J., Standaert, R.F., Engle, N.L., Martin, M.Z., Sangha, A.K., Parks, J.M., Smith, J.C., Samuel, R., Jiang, N., Pu, Y.Q., Ragauskas, A.J., Hamilton, C.Y., Fu, C.X., Wang, Z.Y., Davison, B.H., Dixon, R.A. and Mielenz, J.R. (2012) Down-regulation of the caffeic acid O-methyltransferase gene in switchgrass reveals a novel monolignol analog. *Biotechnol Biofuels* **5**.

Figure S1. GO enrichment of genes co-expressed with *PtPFD2.2*.

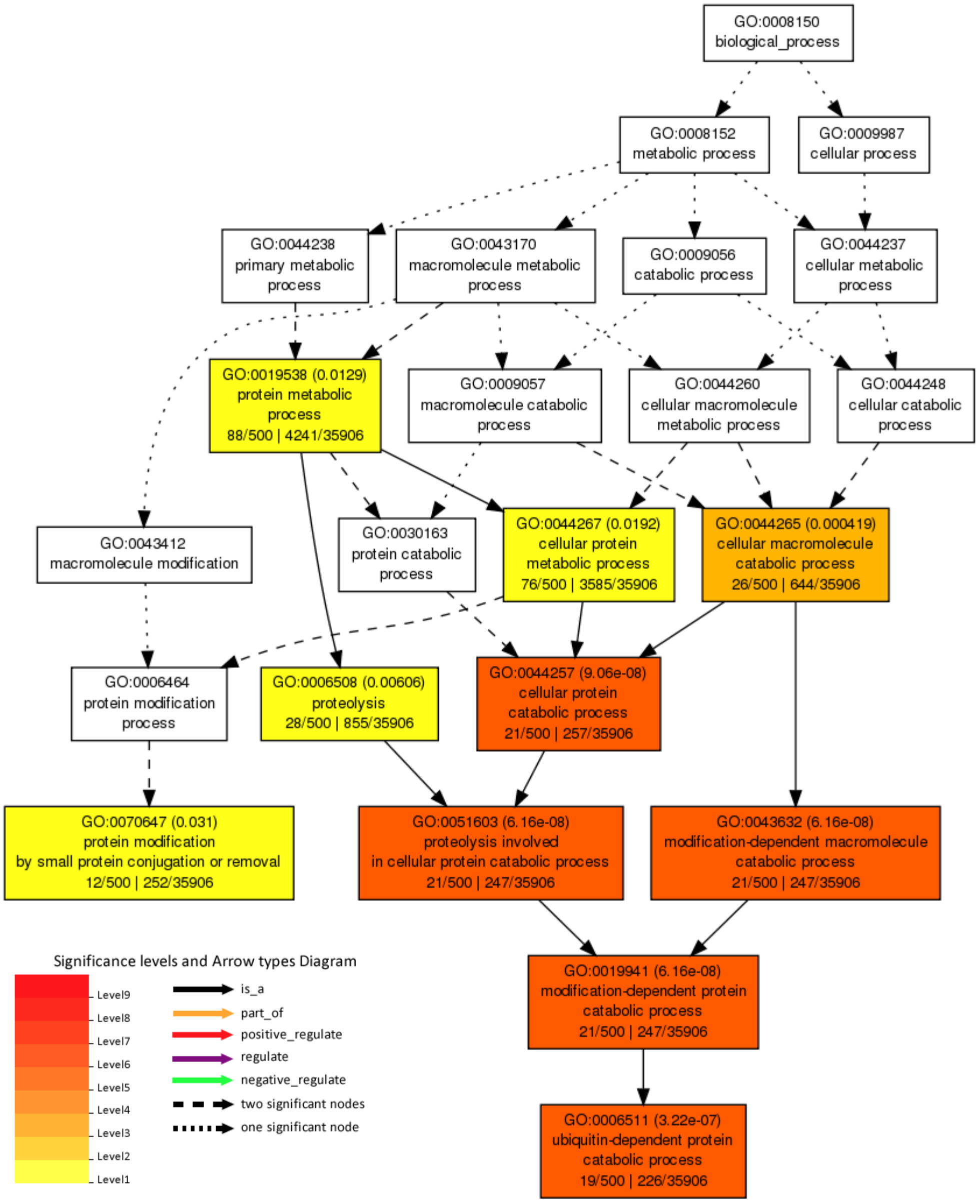

Figure S2. *cis*-acting elements in promoter region of *PtPFD2.2*.

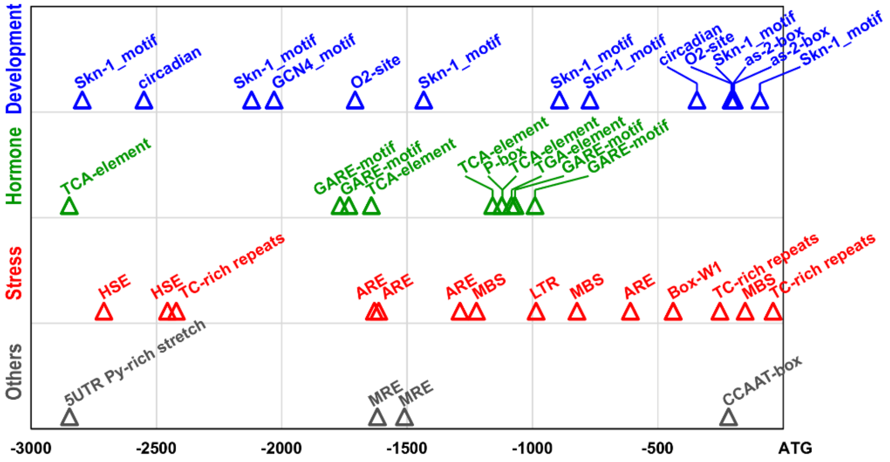

Classification	*cis*-acting elements	Annotation	No.
Development	Skn-1_motif	required for endosperm expression	7
	O2-site	involved in zein metabolism regulation	2
	circadian	involved in circadian control	2
	as-2-box	involved in shoot-specific expression and light responsiveness	2
	GCN4_motif	involved in endosperm expression	1
Hormone	GARE-motif	gibberellin-responsive element	4
	TCA-element	involved in salicylic acid responsiveness	4
	P-box	gibberellin-responsive element	1
	TGA-element	auxin-responsive element	1
Stress	ARE	essential for the anaerobic induction	4
	TC-rich repeats	involved in defense and stress responsiveness	3
	MBS	MYB binding site involved in drought-inducibility	3
	HSE	involved in heat stress responsiveness	2
	Box-W1	fungal elicitor responsive element	1
	LTR	involved in low-temperature responsiveness	1
Others	MRE	MYB binding site involved in light responsiveness	2
	5UTR Py-rich stretch	conferring high transcription levels	1
	CCAAT-box	MYBHv1 binding site	1

Figure S3. Protein structure of PtPFD2.2.

(a) Secondary protein structure of PtPFD2.2 with potential post-translation modification sites. Yellow, red and blue pins represent phosphorylation, sumoylation, and ubiquitination sites, respectively. Conserved Prefoldin_2 motifs identified from pfam database is shown in the blue box (13-118 aa). (b) Protein 3D structure of PtPFD2.2

(b) Protein 3D structure of PtPFD2.2.

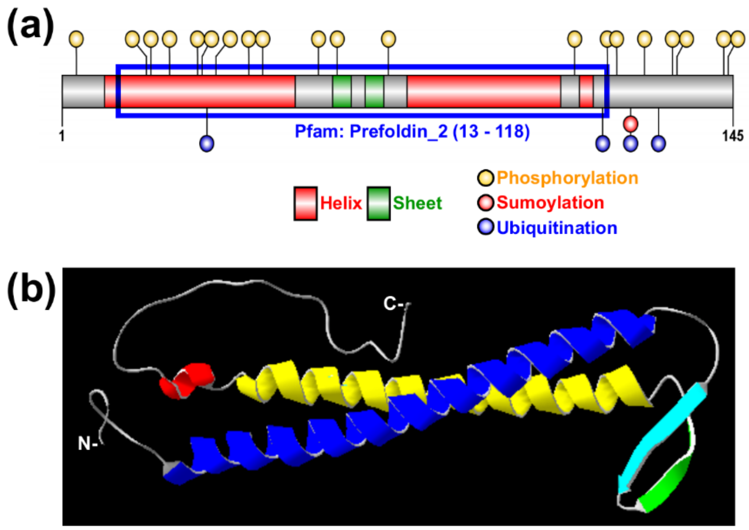

Figure S4. Expression of *PdPFD2.2* in control (Ctrl) and two overexpression lines through qRT-PCR.

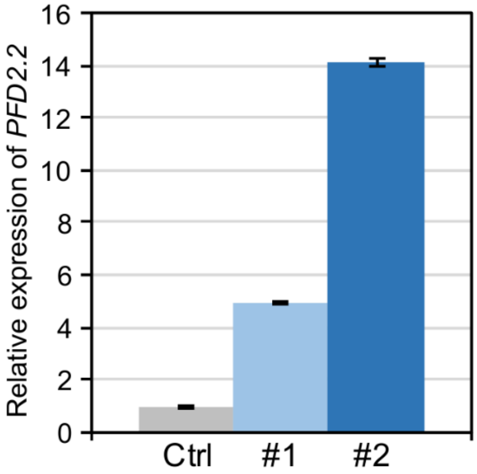

Figure S5. Cellulose and hemicellulose contents and sugar release based on cellulose or hemicellulose in *PdPFD2.2* overexpression lines.

(a) Cellulose content. (b) Hemicellulose content. (c) Glucose release based on the cellulose content. (d) Xylose release based on the hemicellulose content.

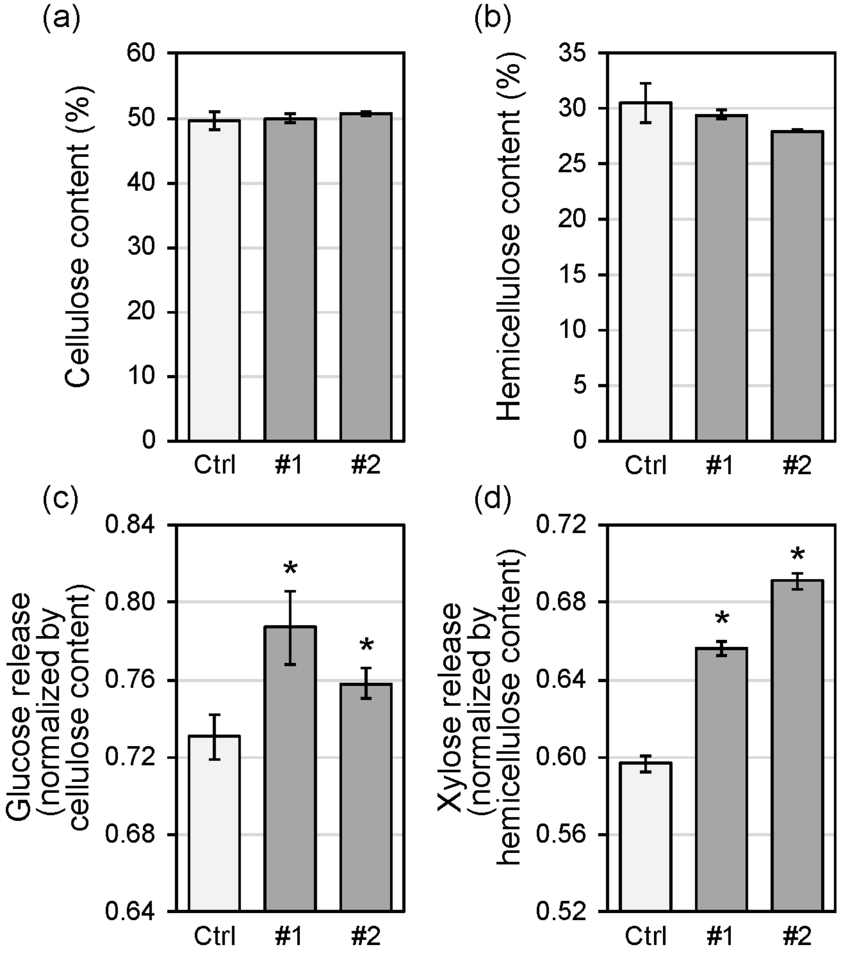

Figure S6. Cellulose crystallinity index (CrI) and degree of polymerization (DP) of cellulose in *Populus* transgenic lines overexpressing *PdPFD2.2*.

(a) CrI measurement using solid-state NMR. (b) The number-average degree of cellulose polymerization (DPn). (c) The weight-average degree of cellulose polymerization (DPw). (d) The polydispersity index (PDI) of cellulose.

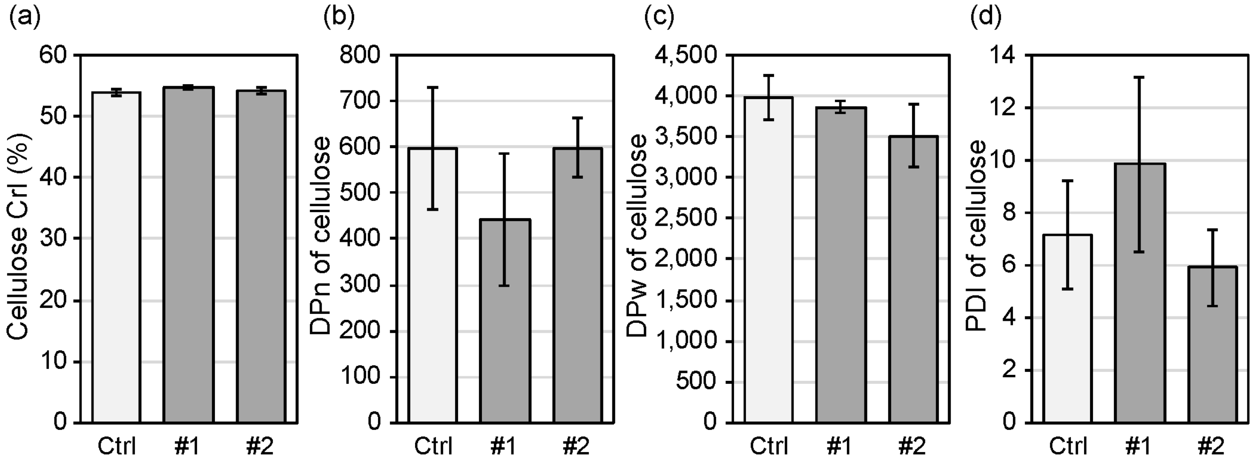

Figure S7. Correlation of biological replicates in RNA-Seq.

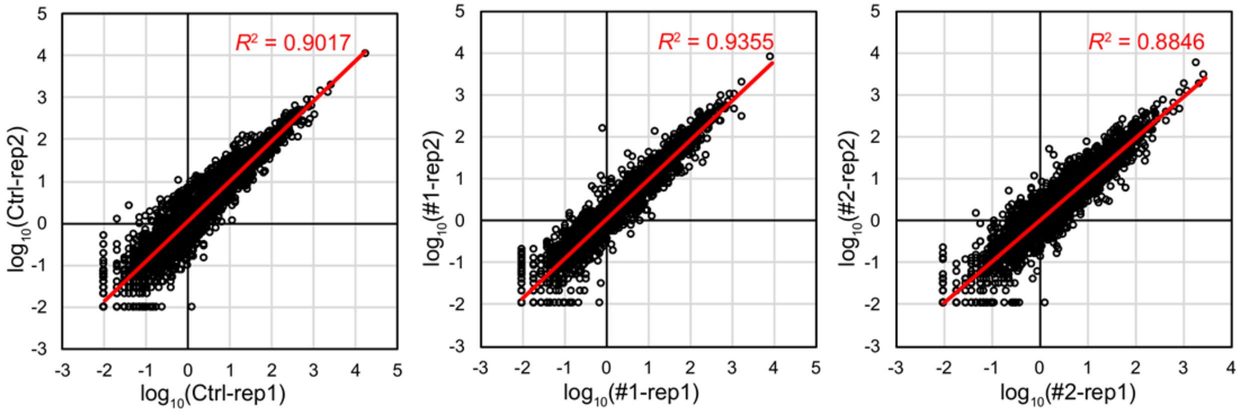

Supplement: Supplementary file 1 — Methods S1 Cellulose and hemicellulose analysis. Methods S2 Saccharification assay. Methods S3 RNA‐Seq analysis. Methods S4 Metabolomic analysis by gas chromatography‐mass spectrometry (GC‐MS). Figure S1 GO enrichment of genes co‐expressed with PtPFD2.2. Figure S2 cis‐acting elements in the promoter region of PtPFD2.2. Figure S3 Protein structure of PtPFD2.2. (a) Secondary protein structure of PtPFD2.2 with potential post‐translation modification sites. Yellow, red and blue pins represent phosphorylation, sumoylation and ubiquitination sites, respectively. Conserved Prefoldin_2 motifs identified from pfam database are shown in the blue box (13–118 aa). (b) Protein 3D structure of PtPFD2.2. Figure S4 Expression of PdPFD2.2 in control (Ctrl) and two overexpression lines through qRT‐PCR. Figure S5 Cellulose and hemicellulose contents and sugar release based on cellulose or hemicellulose in PdPFD2.2 overexpression lines. (a) Cellulose content. (b) Hemicellulose content. (c) Glucose release based on the cellulose content. (d) Xylose release based on the hemicellulose content. Figure S6 Cellulose crystallinity index (CrI) and degree of polymerization (DP) of cellulose in Populus transgenic lines overexpressing PdPFD2.2. (a) CrI measurement using solid‐state NMR. (b) The number‐average degree of cellulose polymerization (DPn). (c) The weight‐average degree of cellulose polymerization (DPw). (d) The polydispersity index (PDI) of cellulose. Figure S7 Correlation of biological replicates in RNA‐Seq analysis. [file PBI-18-859-s002.pdf]
